# Tuning Alginate Microparticle Size via Atomization of Non-Newtonian Fluids

**DOI:** 10.3390/ma14247601

**Published:** 2021-12-10

**Authors:** Beatriz Arauzo, Álvaro González-Garcinuño, Antonio Tabernero, Maria Pilar Lobera, Jesus Santamaria, Eva María Martín del Valle

**Affiliations:** 1 Department of Chemical Engineering and Environmental Engineering, Campus Río Ebro-Edificio I+D, University of Zaragoza, C/Mariano Esquillor S/N, 50018 Zaragoza, Spain; secinma@unizar.es (B.A.); plobera@unizar.es (M.P.L.); jesus.santamaria@unizar.es (J.S.); 2 Networking Research Center on Bioengineering, Biomaterials and Nanomedicine, CIBER-BBN, 28029 Madrid, Spain; 3 Department of Chemical Engineering, University of Salamanca, P/Los Caídos S/N, 37008 Salamanca, Spain; alvaro_gonzalez@usal.es (Á.G.-G.); antaber@usal.es (A.T.)

**Keywords:** alginate, piperazine, barium chloride, air-blast atomization, microparticles

## Abstract

A new approach based on the atomization of non-Newtonian fluids has been proposed to produce microparticles for a potential inhalation route. In particular, different solutions of alginate were atomized on baths of different crosslinkers, piperazine and barium chloride, obtaining microparticles around 5 and 40 microns, respectively. These results were explained as a consequence of the different viscoelastic properties, since oscillatory analysis indicated that the formed hydrogel beads with barium chloride had a higher storage modulus (1000 Pa) than the piperazine ones (20 Pa). Pressure ratio (polymer solution-air) was identified as a key factor, and it should be from 0.85 to 1.00 to ensure a successful atomization, obtaining the smallest particle size at intermediate pressures. Finally, a numerical study based on dimensionless numbers was performed to predict particle size depending on the conditions. These results highlight that it is possible to control the microparticles size by modifying either the viscoelasticity of the hydrogel or the experimental conditions of atomization. Some experimental conditions (using piperazine) reduce the particle size up to 5 microns and therefore allow their use by aerosol inhalation.

## 1. Introduction

A drug delivery system (DDS) can be defined as a formulation that can be administered to a patient’s body and is able to release the drug in a controlled way. The structure of a DDS is often a drug encapsulated inside polymer matrix that acts as a reservoir. The drug is then released from that matrix via different mechanisms, such as diffusion or erosion [1].

Among the characteristics of a DDS, its size is a key parameter that has to be considered in its development because it defines the pathway that has to be followed by the drug to reach the targeted organ. The transdermal route needs particles ranging from 10–600 nm, intravenous 200–2000 nm, and 8–20 microns are required for a nasal dosage and finally, an inhalable aerosol needs particles ranging from 0.1–5 microns [2].

There are many techniques to produce DDS. The choice depends on the final applications and characteristics such as the administration route, the type of drug to be encapsulated, and the desired release rate [3]. Therefore, the technology used to produce the DDS strongly depends on the desirable particle size and the drug dosage form. Moreover, biocompatible and biodegradable materials must be used to produce the polymeric matrix to avoid side effects after DDS administration. In this context, polysaccharides fulfill all the required characteristics [4]. Among the polysaccharides, alginate, which is a linear and anionic polysaccharide with D-mannuronic acid and l-guluronic acid, has been widely used for biomedical purposes [5,6].

One of the most used approaches to produce alginate DDSs is based on the polysaccharide crosslinking (ionotropic gelation) due to its polyelectrolytic behavior. This technique takes advantage of the hydrogel formation by a crosslinking process when an aqueous solution of alginate is put in contact with a solution of a divalent ion [7]. As a consequence, a hydrogel is formed due to the bonding between the divalent cations and the carboxyl groups of the guluronic acid. This gelation mechanism follows the egg-box model, which has been extensively studied in literature [8,9].

In laboratory studies, the alginate solution is often added drop wise on a bath containing the cations. In this way, hydrogel beads-like capsules (of millimetric size) will be formed [10]. This methodology has been widely used to produce DDSs with alginate, and different experimental conditions, such as surface tension, viscosity of both alginate solution and the bath, nozzle diameter, alginate flow rate, and even penetration depth in the bath, can play an important role in particle size and shape. In this context, Davarci et al. [11] used a high-speed camera to study the effect of the previous parameters on the aforementioned process, showing that alginate flow rate and nozzle size were the main parameters that must be controlled to modify the size, whereas the penetration depth is crucial for tuning beads’ sphericity. This work also indicates that viscosity of the alginate solution has a significant effect on both properties, although without a high impact concerning size (always in the millimeter range).

However, millimetric particles face major drawbacks for their future use in pharmaceutical applications because they cannot be used in inhaled aerosols or in transdermal routes [12]. A suitable strategy to reduce particle size with atomization techniques involves creating instability on the liquid flow that emerges from the nozzle. This enhances the jet breakup phenomenon, reducing the droplet size and the final particle size.

In particular, due to the pseudoplastic character of the alginate solutions, the kinetic energy must be increased to achieve a proper jet breakup of these polymeric solutions. Therefore, special designs (referring to nozzle configuration or the equipment) have been proposed in different articles. As an example, an air-blast atomization was used to create a flow of highly viscous solutions of alginate on barium chloride, controlling the different jet breakup modes (from Rayleigh breakup to atomization), obtaining particles around 30 microns [13]. The process was theoretically studied in terms of the most important dimensionless numbers (Weber and Ohnesorge) regarding jet breakup, to verify the effect of the viscosity and surface tension forces [8]. Films of alginate with barium chloride were also produced and used in tissue regeneration without showing toxicity towards healthy cells [14].

Another promising strategy could be the induction of jet disturbances after the jet emerges from the nozzle. This can be achieved by imposing a vibration with a piezoelectric device in the nozzle configuration. This technology allowed the production of particles alginate-barium chloride from 300 to 700 microns, and the work also clearly showed that viscosity control is essential to obtain correct values of polydispersity in the final particle size distribution [15].

Since particles smaller than 30 microns were not obtained with the previous techniques, more strategies were developed to reach the micro and even the nanoscale. Among them, new types of reactions based on chemical interactions addressed to alginate functional groups were proposed.

Specifically, nanoparticles of about 300 nm were obtained with the system alginate-piperazine. This combination of chemicals takes advantage of the molecular structure of the piperazine, which has two amino groups in opposite positions of the molecular ring. Under acidic conditions, these groups get a positive charge, which is attracted to the carboxylic groups of the alginate facilitating reactions to form particles. This structure is different from that obtained with the egg box mechanism [16]. Moreover, these nanoparticles were not toxic towards healthy cells, and they were also subsequently bioconjugated in their surface with epidermal growth factor and cisplatin to produce novel nanocarriers, targeting preferentially cancer cells [16]. This technique was also used in another work, where two drugs, trastuzumab and paclitaxel, were bound to these nanoparticles for treating successfully breast cancer cells HER2+ [17].

However, the previous particles were obtained using batch processes, whose yields are low and reproducibility is also difficult due to the intrinsic characteristics of batch processing [6]. This can be considered a shortcoming to produce the particles at a larger scale. In this regard, the scaling-up is always challenging and has an unknown impact on the DDS, often increasing the average particle size and its polydispersity, which would be negative for a potential application.

In this work, we propose the production of alginate-piperazine particles in a fan-jet nozzle to solve the previous drawbacks. The objective of the study is twofold: to obtain particles smaller than 30 microns and to assess how the particle size is modified after changing the regime, from a discontinuous process to a semicontinuous one. The effect of the different parameters on the particle size, such as an air/solution pressure ratio, concentration, and pseudoplasticity of the alginate solutions will be studied. In addition, a numerical study, calculating the Ohnesorge and Weber modules, will be performed in order to define a semiempirical model to predict the particle size.

Finally, the results with alginate-piperazine particles will be compared with alginate-barium chloride particles (electrostatic interactions vs. gelation egg-box model) that were obtained with the same atomization equipment and with the same nozzle configuration. Therefore, this work proposes as a main novelty the study of an atomization device to produce capsules of a new system, alginate-piperazine, in a semicontinuous way, providing the possibility of tuning particle size, and as a consequence their final application, depending on the used technique and crosslinker as well as the experimental conditions.

## 2. Materials and Methods

### 2.1. Materials

Alginic acid sodium salt from brown algae (medium viscosity), piperazine (99%), calcium chloride (<99%), and sodium hydroxide (98%) were purchased from Sigma Chemicals (Madrid, Spain). Barium chloride dehydrate (>99%), reagent grade was supplied by Scharlau. Hydrochloric acid (37%) was purchased from Panreac (Castellar del Valles, Barcelona, Spain). Air for the atomization system was provided by Air Liquid (99.99%).

### 2.2. Preparation and Characterization of Solutions

Alginate solutions (concentrations from 0.5% *w*/*w* to 2% *w*/*w*) were prepared dissolving alginate sodium salt in distilled water under vigorous stirring (500 rpm) for 12 h at RT.

Piperazine and barium chloride solutions were used as crosslinkers with alginate. Piperazine solutions were prepared at different concentrations (from 0.05% *w*/*w* to 0.15% *w*/*w*). After that, the pH of both solutions was adjusted between 4.6–4.8 with hydrochloric acid (37%) to create an acidic environment that can promote the generation of electrostatic interactions between the carboxylate groups of alginate (pKa values of mannuronic and guluronic acid residues are 3.38 and 3.65, respectively) [18] with the amino groups of piperazine (pKa1 9.73 and pKa2 5.33) [19]. Finally, barium chloride solution was prepared at 2% *w*/*w* in distilled water.

### 2.3. Rheological Experiments

A rheometer AR 1500 ex (TA Instruments, Cerdanyola del Valles, Spain) was used to perform the different rheological measurements. The same geometry was used for all the experiments (an aluminium plate of 4 cm of diameter with a 1 mm gap).

Shear flow experiments were conducted on different solutions of alginate at a temperature of 25 °C. A solution was loaded on the peltier and a shear rate ranging from 0.1 to 250 s^−1^ was selected. After that, the rheological results were fitted to the power law equation (Equation (1)), obtaining the different adjustable parameters (*τ* is the shear stress, *K* is the consistency index, $\dot{\gamma }$ is the shear rate and *n* is the flow behavior index):

(1) $$\tau =K{\dot{\gamma }}^{n}$$

On the other hand, the rheological behavior of (1) alginate-piperazine (Alg-Ppz) and (2) alginate-barium chloride (Alg-BaCl_2_) hydrogels was studied, always at 25 °C, with an oscillatory analysis, performing a frequency sweep from 0.5 to 500 rad·s^−1^ at a fixed strain that must lie in the linear viscoelastic region. This strain was selected after performing previously a strain sweep analysis (from 0.5% to 300% strain) at a constant angular frequency of 1.0 rad·s^−1^ and at 25 °C. According to the strain sweep results, we fixed a 1% strain for the subsequent oscillatory analysis.

Frequency sweep results indicated how strong the interactions are for the different systems, calculating storage modulus (G′(w)) and loss modulus (G′′(w)), and providing information concerning the predominant nature of the hydrogels (solid-like or viscous-like).

### 2.4. Experimental Device and Operational Procedure

**Figure 1:**
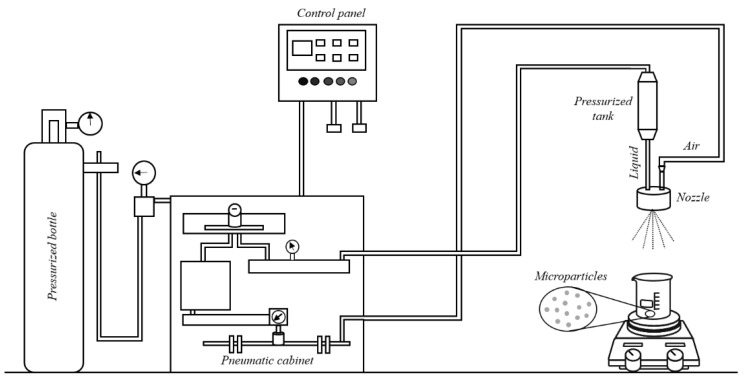
Experimental device to generate microparticles (Adapted from Herrero et al. [8]).

The atomization apparatus was already described elsewhere (Herrero et al.) and is illustrated in Figure 1 [8]. Experiments begin with the introduction of alginate solutions into the pressurized tank (with maximum capacity 50 mL).

After that, air and liquid pressures were adjusted by taking into account that air pressure must be equal or less than liquid pressure. Due to the pressure effect, the alginate solution flows from the tank through a nozzle (inner diameter: 250 µm and outer diameter: 350 µm). On the other part of the nozzle, air is introduced from an air cylinder in order to provide the required kinetic energy and the instabilities to disintegrate the liquid jet. The formed droplets are sprayed into a crystallizer with 140 mm diameter (Scharlab) that contains 300 mL solution of piperazine or BaCl_2_ under magnetic stirring (150 rpm). Once the atomization is finished, the formed microparticles (MPs) were kept for 30 min under stirring in the solutions (piperazine or BaCl_2_) in order to provide enough time to complete the gelation process.

The subsequent particles isolation and purification methodology depend on the system. The piperazine microparticles were collected adapting the method described in Roman et al. [16]. In order to promote particle precipitation due to differences in the pKa, the pH of the solution was set at 1.0 by adding HCl. Later, microparticles were centrifuged at 7500 rpm for 10 min (Eppendorf Centrifuge 5804, Eppendorf, Hamburg, Germany). The supernatant was discarded and the pellet was resuspended in 10 mL of distilled water. After that, the pH was increased up to a value of 3.0–6.0 using NaOH 1 M, which is the required pH to ideally solvate the microparticles and stabilize them in the aqueous solution. This pH was selected because, according to a previous study, a pH above 6.0 can break the particles because the piperazine may be deprotonated. Furthermore, a higher polydispersity was observed at high pH values [16].

In the case of the microparticles obtained with barium chloride, they were recovered by vacuum depth filtration (cutoff: 0.2 µm), and then they were washed 10 times with water in order to eliminate the residual Ba^2+^ ions. Finally, particles are resuspended in distilled water [20].

### 2.5. Experimental Conditions

As already explained, particle size and polydispersity are important parameters that have to be controlled for future biomedical applications. Therefore, different experimental parameters were systematically varied to study their effect on particle properties.

Specifically, the effect of the ratio air pressure/liquid pressure (pressure ratio PR) was studied by varying the liquid and air pressure from 0.6 to 1.0 bar. The effect of alginate concentration was studied by atomizing different alginate solutions (30 mL) of varying concentrations over 300 mL of different piperazine solutions to generate alginate-piperazine microparticles (MPs Alg-Ppz). Experimental conditions are collected in Table S1 in Supplementary Information (SI).

Finally, 30 mL of alginate solution with at 2% *w*/*w* concentration were atomized into 300 mL of barium chloride solution at 2% *w*/*w* to prepare alginate-BaCl_2_ microparticles. Table S2 in SI summarizes the experimental parameters to produce alginate-BaCl_2_ microparticles (MPs Alg-BaCl_2_).

In this context, it is necessary to specify that only concentrations of 2% *w*/*w* of alginate and 2% *w*/*w* of BaCl_2_ were considered for the system alginate-barium chloride. As explained in the Results section, polydisperse particles were obtained for different values of viscosity. The results obtained with these microparticles were included here for comparison purposes with alginate-piperazine microparticles.

### 2.6. Characterization of Microparticles

Microparticles were characterized in terms of particle size distribution, surface charge distribution (Zeta potential), and morphology (Optical microscope (LEICA DM1000, Leica Microsystems, Wetzlar, Germany) and Environmental Scanning Electron Microscope (ESEM)).

Zeta potential for both types of microparticles was measured with Zetasizer nano-ZS90 (Malvern, Malvern, UK). Each value was obtained as an average of three measurements.

MPs Alg-Ppz size were measured by dynamic light scattering (DLS) using Zetasizer nano-ZS90 (Malvern, Malvern, UK). In this case, samples should be previously diluted in distilled water and sonicated for 10 min at 28–34 kHz (Starsonic 90, Liarre Digit) previous performing the measurement, which provides particle size distribution (hydrodynamic diameters) as well as the polydispersity index (PDI).

On the other hand, MPs Alg-BaCl_2_, particle size distribution, and PDI were determined using a particle analyzer (Mastersizer2000, Malvern Instruments, Malvern, UK) with laser diffraction technology, obtaining as a result the Sauter Mean Diameter (SMD). Samples were also sonicated for 10 min at 28–34 MHz (Starsonic 90, Liarre Digit) as was done for the DLS.

Image characterization of the particles was done by optical microscope DM1000 Leica (Leica Microsystems, Wetzlar, Germany) with camera Leica DFC280. Camera was calibrated in order to estimate the size of the particles observed by using the software Measure IC (version 2.4.633.2555, Charlotte, NC, USA)

Environmental Scanning Electron Microscope (ESEM) (SEM-Quanta FEG-250, FEI, Hillsboro, OR, USA) was employed to observe microparticles as well. ESEM images were obtained by microparticles in water and observing them at 1 °C and 456 Pa.

### 2.7. Particle Size Distribution Estimation

Particle size distribution results were numerically fitted to a mathematical model. This mathematical model was developed by taking into account the main dimensionless numbers in an atomization phenomenon: Weber (*We*) and Ohnesorge (*Z*). Both numbers are defined in Equations (2) and (3). *We* number expresses the equilibrium between aerodynamic forces and surface tension forces, whereas *Z* number expresses the equilibrium between viscous forces and tension forces:

(2) $$We=\frac{\rho \cdot v^{2}\cdot l}{\sigma }$$

(3) $$Z=\frac{\mu }{\sqrt{\rho \cdot \sigma \cdot l}}$$

where *ρ* is the density of fluid, *v* is the jet velocity, *l* was taken as the nozzle inner diameter, in agreement with [21], *σ* is the surface tension and *µ* is the viscosity of the fluid. The used correlation was developing by Kennedy [22], which is described in Equation (4):

(4) $$\frac{SMD}{l}=a\cdot We^{-b}+c\cdot Z^{b}+d$$

where *SMD* is the Sauter Mean Diameter, and *a*, *b*, *c* and *d* are fitting parameters. Different dimensionless numbers were calculated for each experiment, with a different *SMD*. Therefore, it would be possible to predict microparticles size if the fitting parameters are numerically calculated.

Parameter estimation was carried out in gPROMS 5.0.2 (PSE), applying the previous equations, to the experimental data obtained (performing a Hessian estimation). Data from four different alginate concentrations were used (0.5%; 0.75%; 1.0%; 1.5% *w*/*w*). Constant variance was selected as variance model and the sensor for parameter estimation was fixed as a function of the *SMD* in a range from 1% to 5% *w*/*w*. Four parameters were estimated (*a*, *b*, *c*, *d*) without boundary conditions.

In parallel with parameter estimation, a statistical analysis was carried out where weighted residuals were calculated and compared with chi-square value (χ^2^) for a confidence interval of 95%. If the value of weighted residuals is less than χ^2^ value, it is assumed that the model predicts accurately the experimental data.

Moreover, once parameters were estimated, a consistency test was performed in order to validate the estimation by using outlier’s concentrations of alginate (out of the range used for parameter estimation). Specifically, the alginate concentrations used were 0.4% *w*/*w* and 1.8% *w*/*w*.

## 3. Results and Discussion

### 3.1. Rheological Results

#### 3.1.1. Alginate Solutions

Table 1 shows the density, surface tension, and viscosity of the different alginate solutions. As can be seen in the table, the density of the solutions was almost constant (1 g·cm^−3^), whereas the surface tension values ranged from 0.096 to 0.142 N·m^−1^.

**Figure 2:**
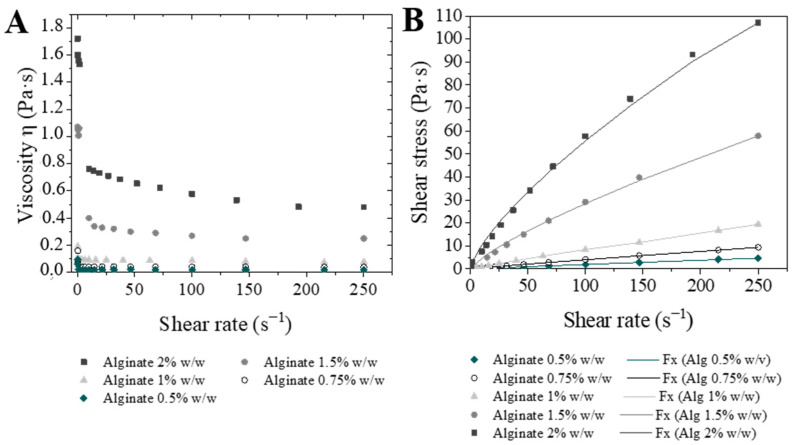
Representation of rheological results: (**A**) Viscosity vs. *γ* (Shear rate); (**B**) *τ* (Shear stress) vs. *γ* (Shear rate).

The highest difference was found for the viscosity of the solutions, ranging from 15 to 450 cP. These values were calculated as the slope of the curve shear stress–shear rate (Figure 2A) that, at the same time, was determined with steady shear experiments. Moreover, Figure 2A,B illustrate the pseudoplastic nature of the alginate. This behavior indicates that at the beginning (without shear rate) the polymeric chains are intertwined and the flow resistance is higher. However, increasing the shear rate promotes a polymer chains alignment, and as a consequence the viscosity is reduced. This phenomenon has been already reported for many polymeric solutions [21,23].

Shear stress-shear rate curve was fitted with a power law equation (Equation (1)). The fitting results (Table 2) indicate that the pseudoplasticity increases with alginate concentration, mainly from a concentration of 1% *w*/*w* (the index flow C decreases from 0.9410 (1% *w*/*w*) to 0.7336 (2.0% *w*/*w*)). The effect of this parameter in the microparticles will be studied in the following sections.

#### 3.1.2. Alginate-Piperazine and Alginate-Barium Chloride

**Figure 3:**
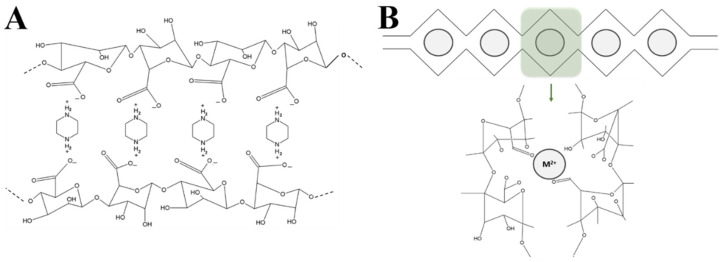
(**A**) Schematic representation of interactions between alginate and piperazine to form microparticles; (**B**) schematic representation of the interactions between alginate and divalent ions.

Figure 3 schematically depicts the different electrostatic interactions between the alginate and piperazine or barium chloride and identifies the functional groups responsible for them. As will be seen below, these interactions determine a very different rheological behavior for both systems. 

**Figure 4:**
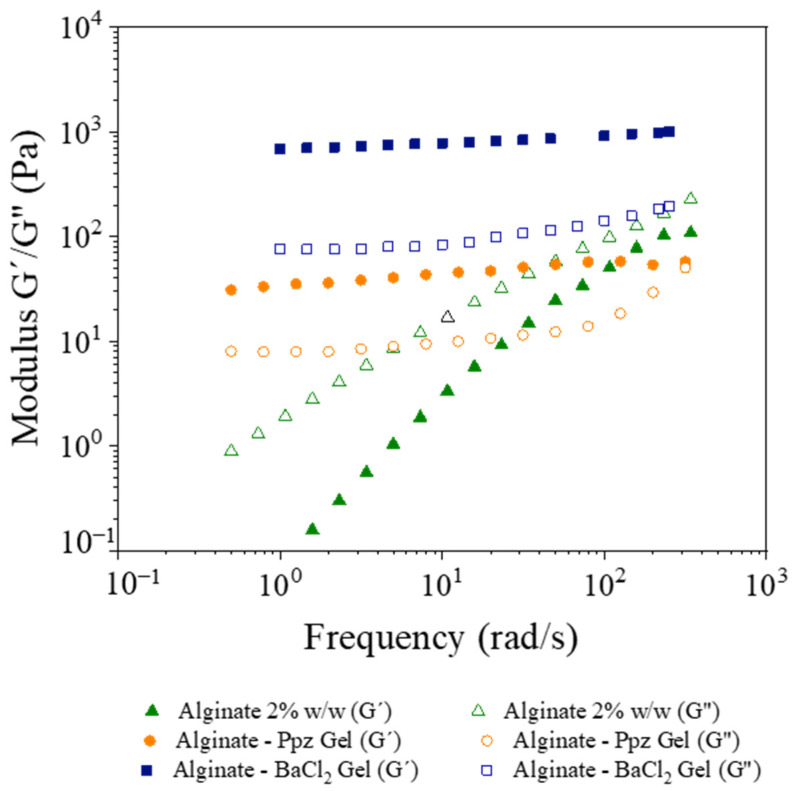
Storage (G′) and viscous (G′′) modulus of alginate solution, Alg-Ppz gel and Alg-BaCl_2_ gel.

Figure 4 shows the rheological results after performing an oscillatory analysis on the alginate-piperazine and alginate-barium chloride gels and on the alginate solutions. It can be seen that the oscillatory analysis of the alginate solutions provides the typical result for a viscous solution, where the loss modulus G′′ is higher than the storage modulus G′, and, as a consequence, the viscous nature predominates.

However, when the alginate solution is put in contact with piperazine or barium chloride, the mechanical spectrum changes. The storage modulus, G′, increases and is always higher than the G′′. This fact indicates the existence of interactions between the alginate chains and the respective compounds (piperazine and barium chloride), as depicted in Figure 3. In this context, the same amount of piperazine or barium chloride solutions with the same concentration (2% *w*/*w*) were used to crosslink alginate solution of 2% *w*/*w*.

The value of the storage modulus slightly increases with the frequency for both systems, indicating the existence of remaining interactions. Moreover, Figure 4 also shows that the difference between G′ and G′′ of the alginate-barium chloride complex is higher (1000 Pa and 70 Pa) than the same difference for the alginate-piperazine system (30 Pa and 10 Pa). Moreover, the curves of the alginate-piperazine intersect at high frequency, highlighting gel weakness. These results indicate that alginate-piperazine gels are weaker than the alginate-barium chloride gels and, as a consequence, their viscoelastic properties are different.

### 3.2. Comparison in Production of Both Types of Microparticles

#### 3.2.1. Influence of Liquid and Air Pressure

In order to identify the required pressures to obtained microparticles, air and liquid pressures were modified to obtain a pressure map, which will indicate if the pressure ratio, PR, has an impact on particle size.

Previous studies in the research group [9] showed that a minimum value of air and liquid pressure is necessary to achieve an atomization regime. Air pressure cannot be greater than liquid pressure because, then, air would enter inside the nozzle and would prevent the liquid to emerge. Therefore, the PR, defined as the quotient between air and liquid pressure, cannot be higher than 1. However, if air pressure is significantly lower than liquid pressure, the air would not have enough kinetic energy to produce the jet breakup. When the value of the air pressure is similar to the value of the liquid pressure, the aerodynamic forces are able to overcome capillary and viscosity forces, producing alginate solutions’ atomization.

**Figure 5:**
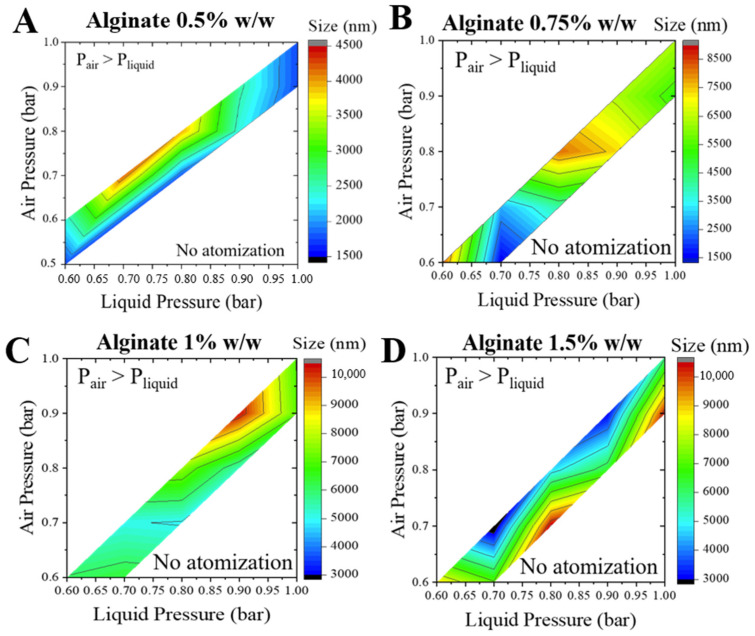
Alg-Ppz MPs. Air and liquid pressures influence on particle size diameter for different alginate concentrations; (**A**) 0.5% *w*/*w*; (**B**) 0.75% *w*/*w*; (**C**) 1.0% *w*/*w*; (**D**) 1.5% *w*/*w*.

**Figure 6:**
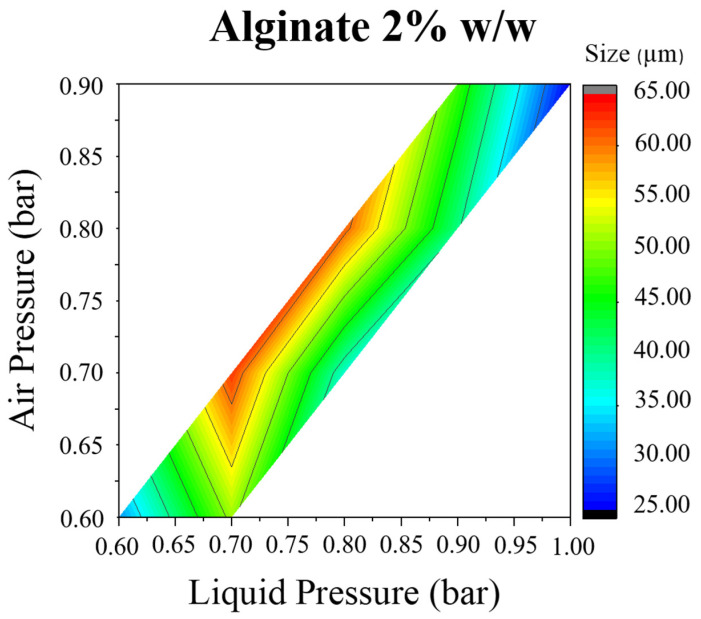
Alg-BaCl_2_ MPs. Air and liquid pressure influence on particle size diameter for 2% *w*/*w* alginate.

As described above, different structures were obtained for each particle due to different interactions (Figure 3). For that reason, the obtained pressure maps were also different. According to the results, the liquid minimum pressure should be around 0.6 bar, whereas air pressure should have a minimum value of 0.5 bar in order to achieve atomization. These pressures ensured the jet break up and microparticles formation. From these experiments, it is possible to conclude that the minimum value of PR to produce atomization, for alginate-piperazine and alginate-barium chloride, is around 0.85 (Figure 5 and Figure 6).

For the system with piperazine as a crosslinker, it is possible to observe the effect of the alginate concentration on the particle size (Figure 5). From 0.5% *w*/*w* to 1.0% *w*/*w*, the bigger microparticles were produced at intermediate values of air and liquid pressure. However, solutions of alginate with a concentration of 1.5% *w*/*w* formed an agglomeration of particles with an elongated shape in the liquid surface due to the high viscosity, and the pressure map was clearly different.

Similar results were obtained when a solution of 2% *w*/*w* of barium chloride was used as a crosslinker for an alginate solution of 2% *w*/*w*. The biggest particles were obtained when air and liquid pressure had intermediate values. When air and liquid pressure were close to 1 at high and low pressures, particles had a small size (Figure 6).

Since the pressure maps for the alginate-piperazine system were markedly different depending on the alginate concentration, a subsequent study was performed to identify the effect of the viscosity of the alginate solutions on the particle size.

#### 3.2.2. Viscosity Influence

The viscosity of the alginate solution plays an important role in the atomization process, as already observed in the pressure maps.

In particular, Table 3 shows the effect of the viscosity on the alginate-piperazine particle size. According to the results, for a fixed pressure ratio, particle size increases with viscosity up to an alginate concentration of 1.0%. At this concentration, the solutions become highly pseudoplastic (according to the Power Law fit in Table 2) and the effect of viscosity on particle size becomes negligible. Here, it should be specified that agglomerated and elongated particles were found on the bath surface for a viscosity higher than 250 cP. This phenomenon can be explained due to the strong pseudoplasticity of the solutions and the increase of the surface tension that can prevent an optimum jet breakup to produce microparticles with piperazine as a crosslinker.

Therefore, ideal and more Newtonian solutions are preferred to obtain smaller alginate-piperazine particles, although it should be considered that highly diluted alginate solutions cannot be crosslinked with piperazine due to the lack of enough carboxylic groups in the polymer.

On the other hand, different results were found for the system alginate-barium chloride. Semi-broken and irregular particles were obtained for alginate solutions up to 90 cP (data not show), and this result was also observed by Mazzitelli et al. [24]. Taking this into account, a concentration of 2% *w*/*w* was selected for the experiments for the alginate-barium chloride.

The different results between the two systems can be attributed to the variations in the gelation mechanisms and the internal structure of the formed beads. Higher viscosity solutions are more adequate to produce particles with an atomization process if the interactions polymer-crosslinker are electrostatic and weak (alginate-piperazine) and with a low storage modulus. However, high concentrations can produce agglomerated particles due to the large number of interactions that are established between the amino groups and the polymer.

On the other hand, low concentration alginate solutions cannot be suitable to produce particles when the formed gels are stronger with a higher storage modulus (alginate-barium chloride). In this case, in spite of the reduction of the surface tension, the egg-box gelation model required higher concentrations of alginate for performing a proper crosslinking than can be achieved with this atomization system.

### 3.3. Microparticles Size and Morphology

Based on the previous results (Section 3.2.1 and Section 3.2.2), the experimental conditions concerning polymer concentration and pressure ratio (PR) were selected to obtain microparticles of the different systems.

Specifically, 1% *w*/*w* alginate solution was selected to produce Alg-Ppz MPs in order to ensure a sufficient polymer concentration able to crosslink with piperazine. In contrast, the Alg-BaCl_2_ MPs were generated from a fixed 2% *w*/*w* alginate solution. Moreover, according to the pressure maps, the selected PR was from 0.85 to 1.

However, there were significant differences in the particle size between alginate-piperazine and alginate-barium chloride microparticles (Barium chloride microparticles are approximately 7-fold bigger than piperazine ones). These differences were caused by the interactions between the polymer and the crosslinker for both systems. In this context, Table 4 and Table 5 show size and polydispersity for the different microparticles.

**Figure 7:**
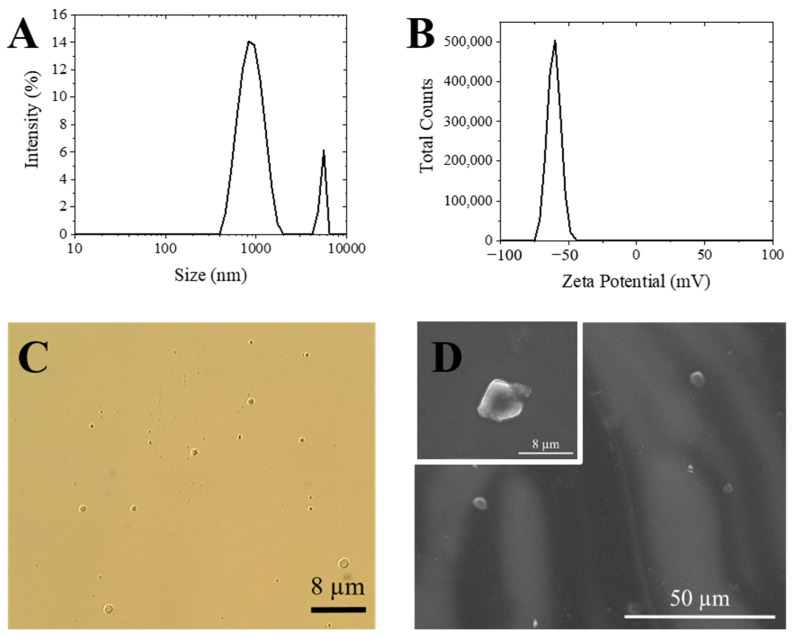
Characterization of Alg-Ppz MPs. (**A**) Size distribution by DLS; (**B**) zeta potential by DLS; (**C**) optical microscope image; (**D**) ESEM images.

Table 4 refers to the particle size (mean Z value) and the polydispersity index (PDI) of Alg-Ppz MP obtained by DLS. The mean diameters of the particles in most of the formulations ranged between 4.50 µm and 10 µm. The PDI of the samples obtained was lower for the Ppz 4 (0.351) and Ppz 5 (0.398). These experiments were performed at the best experimental conditions in the pressure map (Figure 5C). Figure 7A shows a typical PSD for this system, which is characterized by two peaks. The main peak is around 2 µm, with a small amount of aggregates with a size less than 6 µm.

**Figure 8:**
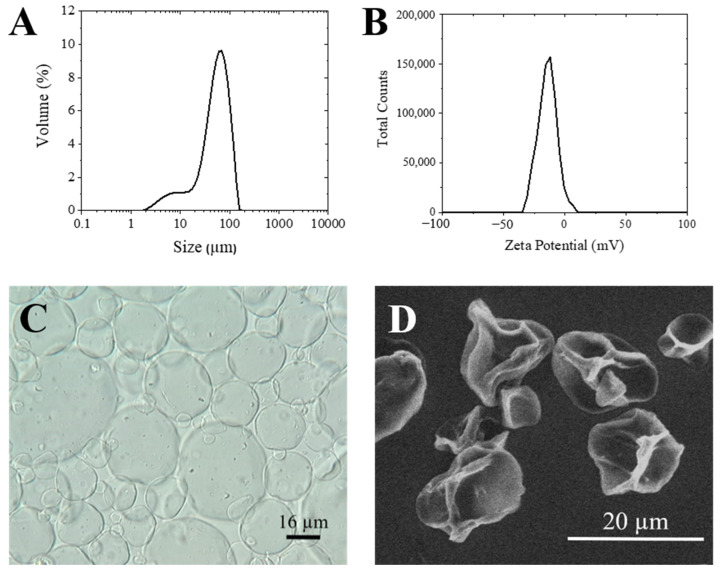
Morphological characterization of Alg-BaCl2 MPs. (**A**) size distribution by Mastersizer; (**B**) zeta potential by DLS; (**C**) optical microscope image; (**D**) ESEM image.

On the other hand, bigger microparticles (from 30 to 65 µm) were obtained when alginate was crosslinked with barium ions. These results agree with the literature, where the smallest particles that have been obtained for this system had a size around 30 microns [16,17,25,26]. The mean size obtained among all experiments was 45 µm. The distribution width (Span) ranged from 1.586 to 4.580, which indicates significant particle size variation in the same formulation (Table 5). It is important to specify that the best results concerning particle size (30 µm) and Span (1.586) were obtained at high pressures according to the pressure map (Figure 6). In most cases, the distribution showed a predominant size close to 60–80 µm (d_0.5_) and another group of particles with smaller sizes (10–20 µm) (d_0.1_) (Figure 8A).

These results provide us the possibility of tuning the particle size depending on the required application. The nanoparticles obtained with piperazine are good candidates if pulmonary delivery the target, given the size range obtained [27]. However, for other applications requiring a larger size, for example mucosal delivery [28], barium chloride is more suitable as a crosslinker agent. The Z potential had a negative value on both particles. The particles generated with piperazine were measured in aqueous medium (pH 6–7), which made both the alginate and part of the piperazine deprotonated, giving high zeta potential values (−40.7 mV (Figure 7B)). In the case of microparticles obtained with barium, it is important to notice that only the carboxylic acid of guluronic acid reacts with the barium in the gelling process. Therefore, the remaining carboxylate groups are responsible for the negative charge of the particles. The obtained value was −8.60 mV (Figure 8B).

Finally, a small amount of particles suspended in water was observed both by optical microscopy and by ESEM. The images obtained by microscopy allowed us to observe the morphology of the generated particles, as well as to confirm their size. The size of the Alg-Ppz microparticles ranged from 0.5 to 4 µm, which agreed with the values obtained by DLS (Figure 7). On the other hand, as expected, the MPs of Alg-BaCl_2_ showed a size greater than previous ones (30–40 µm). The ESEM images of the Alg-BaCl_2_ particles showed a rough surface due to the drying process during sample preparation, which caused the dehydration of the particles (Figure 8). The images taken in both microscopes confirmed the spherical morphology of both particles.

### 3.4. Mathematical Modeling of the Alginate-Piperazine System

A mathematical model was built based on the previous experimental results to predict the particle size as a function of the different experimental conditions. Furthermore, the model will provide an additional theoretical basis to explain the effect of the different parameters in the experimental results. The model was developed for the alginate-piperazine system, for which a wider range of experimental parameters had been studied.

#### Alginate-Piperazine Microparticles

According to materials and methods section, dimensionless numbers were calculated for each atomization experiment with piperazine as crosslinker agent. Table 6 shows the value of *We* and *Z* numbers for each experiment, the concentration (% *w*/*w*) of the alginate solution, and the average size diameter (*SMD*) obtained by DLS measurements (Section 3.3).

From Table 6, it is possible to observe how the increase in Ohnesorge number (*Z*) increases the *SMD* up to a value of around 6–7 µm. Therefore, the particle size is mainly controlled by polymer solution viscosity, which is the main resistance to jet break.

**Figure 9:**
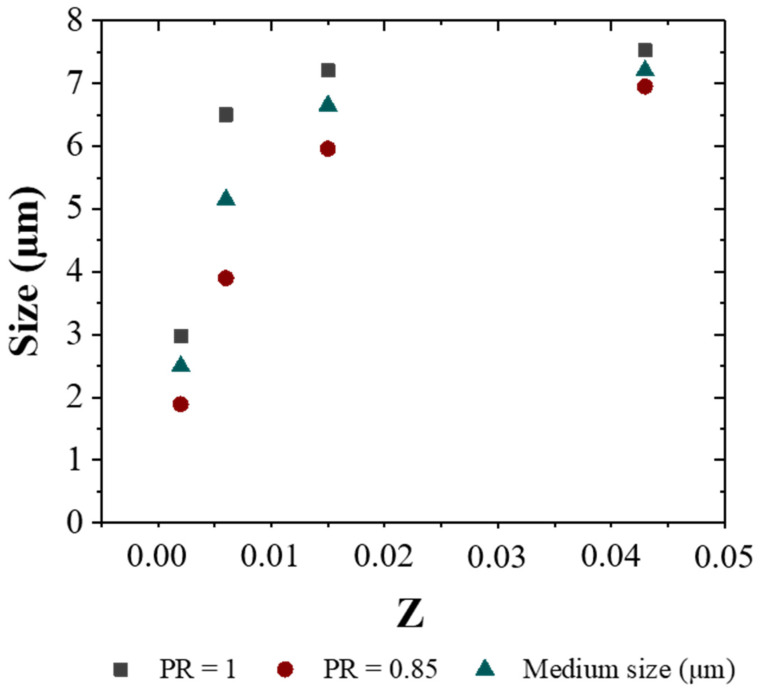
Influence of the Ohnesorge number on size particle.

Moreover, results in Table 6 indicate that there is an important reduction in size if pressure ratio comes from 1.0 to 0.85 (also illustrated in Figure 9), and this reduction is more significant for lower alginate concentrations.

Table 6 shows also that the variation of Ohnesorge number is more significant (the value is multiplied up to 20 times) than the variation in the Weber number (from 2600 to 3100) for all the investigated conditions. From these data, parameters *a*, *b*, *c*, and *d* in Equation (4) were estimated.

These parameters were obtained by Hessian estimation, as described in Section 2.7, using the results for PR equal to 1 and results for PR around 0.85. The estimated values (*a*, *b*, *c*, and *d*) are summarized in Table 7 as well as the estimation time, number of iterations, value of weighted residuals, and value of chi-square (χ^2^).

The value of the weighted residuals was always less than the chi-square value for a confidence level of 95%. Therefore, it is possible to conclude that Hessian estimation was performed effectively with statistical significance.

Although there are differences in the parameters depending on the PR, some similarities can be observed: the quotient between parameters *a* and *c* is near (−1) in both cases. Moreover, there is no significant difference in b parameter, and the main difference among two simulations is the value of parameter d (independent term).

The consistency test confirms that the estimated parameters can be used to predict the particle size depending on the alginate concentration and the PR. According to the methodology section, two additional experiments were performed with concentrations out of the range used for simulation (0.4% and 1.8% *w*/*w*), and for both PR (1 and 0.85). Table 7 summarizes these results and include the SMD estimation based on the parameters previously reported. It can be observed that the model is able to predict the particle size satisfactorily (Table 8), even when the experimental conditions are out of the studied range.

## 4. Conclusions

This study concerning the formation of alginate microparticles via a semicontinuous atomization technique shows the possibility of selecting particle sizes in two different ranges depending on the used crosslinker. Specifically, particles with diameters ranging from 5 to 40 microns were obtained by modifying the gelation mechanism. This is a significant step towards achieving strict control on particle size, one of the challenges in this field.

The rheological study has shown the formation of gels with both crosslinkers, a weaker gel with piperazine and a stronger gel with barium chloride. The study of the values of the pressure maps and the viscosity of alginate solutions has made it possible to establish a relationship between the size of the particle and the experimental conditions used.

Finally, the mathematical model developed allowed us to establish a relationship between the experimental variables and the data obtained with the proposed model. The particle size obtained with an atomization system can be controlled depending on the experimental variables, notably the viscosity, surface tension, and jet velocity grouped in the Weber and Ohnesorge numbers, together with the nozzle design diameter. This work highlights how an atomization device can be used as a semicontinuous technique and how in this technique a different crosslinker agent and the alginate concentration can modify the particle size to the ranges needed according to the intended biomedical application.

## Figures and Tables

**Table 1:** Properties of the alginate solutions.

Alginate (% *w*/*w*)	Density (g/cm^3^)	Surface Tension (N/m)	Viscosity (cP)
0.50	0.999	0.096	15
0.75	1.001	0.102	40
1.00	1.004	0.111	90
1.50	1.006	0.115	250
2.00	1.008	0.142	450

**Table 2:** Pseudoplastic parameters of alginate solutions.

Alginate (% *w*/*w*)	*K* (Pa·s)^n^	n	Standard Error
0.50	0.015	0.9642	2.65
0.75	0.038	0.9246	4.66
1.00	0.089	0.9410	5.63
1.50	0.250	0.7812	10.23
2.00	1.901	0.7336	18.49

**Table 3:** Effects of the viscosity on the Alg-Ppz MPs size.

Pressure Ratio	Alginate Concentration (%)	Viscosity (cP)	Size (µm)
1	0.50%	15	2.969
	0.75%	40	6.401
	1.00%	90	7.203
	1.50%	250	6.951
0.85	0.50%	15	1.898
	0.75%	40	4.000
	1.00%	90	5.956
	1.50%	250	6.000

**Table 4:** Particle size of alginate (1% *w*/*w*) particles with piperazine as crosslinker.

Microparticles		Z-Average Size (µm)	PDI	Flow (L/h)	Pressure Ratio
Formulations	Ppz 1	6.04 ± 0.50	0.818	5.4	1
	Ppz 2	6.37 ± 0.60	0.456	5.8	0.85
	Ppz 3	5.06 ± 0.42	0.470	5.8	1
	**Ppz 4**	**4.93 ± 0.69**	**0.351**	**6.1**	**0.85**
	Ppz 5	7.56 ± 1.99	0.398	6.1	1
	Ppz 6	5.42 ± 0.19	0.535	6.5	0.85
	Ppz 7	10.41 ± 1.27	0.510	6.5	1
	Ppz 8	7.11 ± 0.27	0.482	6.7	0.85
	Ppz 9	6.94 ± 0.84	0.430	6.7	1

**Table 5:** Particle size of alginate particles with barium chloride as crosslinker.

Microparticles		SMD (µm) (3,2)	Span	d_0.1_ (µm)	d_0.5_ (µm)	d_0.9_ (µm)	Flow (L/h)	Pressure Ratio
Formulations	BaCl_2_ 1	30.75	2.334	20.30	43.90	122.95	5.4	1
	BaCl_2_ 2	50.90	3.154	32.95	74.87	269.07	5.8	0.85
	BaCl_2_ 3	62.60	1.892	27.70	211.90	428.70	5.8	1
	BaCl_2_ 4	37.56	2.020	23.55	58.75	141.60	6.1	0.85
	BaCl_2_ 5	60.94	4.580	27.60	84.74	415.75	6.1	1
	BaCl_2_ 6	40.45	2.168	24.70	64.75	165.09	6.5	0.85
	BaCl_2_ 7	47.38	2.747	23.26	149.61	434.28	6.5	1
	BaCl_2_ 8	**41.05**	**1.586**	**26.48**	**60.81**	**112.02**	**6.7**	**0.85**

**Table 6:** Dimensionless numbers for each experiment and their particle size associated.

Pressure Ratio	Alginate Concentration (%)	*Z*	*We*	*SMD* (µm)
1	0.50%	0.002	3105	2.969
	0.75%	0.006	2920	6.401
	1.00%	0.015	2705	7.203
	1.50%	0.043	2610	6.951
0.85	0.50%	0.002	3105	1.898
	0.75%	0.006	2920	4.000
	1.00%	0.015	2705	5.956
	1.50%	0.043	2610	6.000

**Table 7:** Results of parameter estimation.

		PR: 0.85	PR: 1.0
**Parameters estimated**	*a*	−7.125	−7.92
	*b*	8.85 × 10^−4^	8.01 × 10^−4^
	*c*	7.44	8.11
	*d*	−0.312	−0.1873
**Calculous time (s)**		74	102
**Number of iterations**		281	386
**Weighted residuals**		891.4	415.7
**χ** ^2^		99,999	99,999

**Table 8:** Results of consistency test.

Alginate Concentration	PR	Experimental (µm)	Estimated (µm)
0.4% (*w*/*w*)	0.85	0.768	0.957
	1	1.111	2.127
1.8% (*w*/*w*)	0.85	7.469	9.614
	1	8.741	8.340

## Data Availability

The data presented in this study are available on request from the corresponding author. The data are not publicly available due to intellectual property concerns.

## Supplementary Materials

The following are available online at https://www.mdpi.com/article/10.3390/ma14247601/s1, Table S1: Experimental conditions to MPs Alg-Ppz, Table S2: Experimental conditions to MPs Alg-BaCl_2_.

## Nomenclature

G′(w)	storage modulus
G′′(w)	loss modulus
*K*	consistency index
*l*	nozzle inner diameter
*n*	flow behavior index
*v*	velocity of the jet
*We*	Weber number
*Z*	Ohnesorge number
**Greek letters**	
*µ*	viscosity of the fluid
*ρ*	density of the fluid
*σ*	surface tension
*τ*	shear stress
$\dot{\gamma }$	shear rate
