# Supplementary material for: Tuning Alginate Microparticle Size via Atomization of Non-Newtonian Fluids
Source: Materials (Basel). 2021 Dec 10;14(24):7601. doi: 10.3390/ma14247601 (PMC8709414; doi:10.3390/ma14247601)
Supplement: Supplementary file 1 [file materials-14-07601-s001.zip › materials-1499578-supplementary.pdf]

SUPPLEMENTARY MATERIAL

Table S1. *Experimental conditions to MPs Alg – Ppz*

	**Alginate (w/w %)** (30 mL)	**Piperazine (w/w %)** (300 mL)	**Flow (l/h)**	**PR***
Formulations	0.5	0.05	5.4	0.85
	0.5	0.05	5.4	1
	0.5	0.05	5.8	0.85
	0.5	0.05	5.8	1
	0.5	0.05	6.1	0.85
	0.5	0.05	6.1	1
	0.5	0.05	6.5	0.85
	0.5	0.05	6.5	1
	0.5	0.05	6.7	0.85
	0.5	0.05	6.7	1
	0.75	0.075	5.4	0.85
	0.75	0.075	5.4	1
	0.75	0.075	5.8	0.85
	0.75	0.075	5.8	1
	0.75	0.075	6.1	0.85
	0.75	0.075	6.1	1
	0.75	0.075	6.5	0.85
	0.75	0.075	6.5	1
	0.75	0.075	6.7	0.85
	0.75	0.075	6.7	1
	1	0.1	5.4	1
	1	0.1	5.8	0.85
	1	0.1	5.8	1
	1	0.1	6.1	0.85
	1	0.1	6.1	1
	1	0.1	6.5	0.85
	1	0.1	6.5	1
	1	0.1	6.7	0.85
	1	0.1	6.7	1
	1.5	0.15	5.4	0.85
	1.5	0.15	5.4	1
	1.5	0.15	5.8	0.85
	1.5	0.15	5.8	1
	1.5	0.15	6.1	0.85
	1.5	0.15	6.1	1
	1.5	0.15	6.5	0.85
	1.5	0.15	6.5	1
	1.5	0.15	6.7	0.85
	1.5	0.15	6.7	1

*PR: (Air Pressure/Liquid Pressure)

Table S2. *Experimental conditions to MPs Alg – $BaCl_2$*

	Alginate (w/w %)	***Barium chloride (w/w %)***	***Flow (L/h)***	***PR****
	(30 mL)	(300 mL)		
Formulations	2	2	5.4	1
	2	2	5.8	0.85
	2	2	5.8	1
	2	2	6.1	0.85
	2	2	6.1	1
	2	2	6.5	0.85
	2	2	6.5	1
	2	2	6.7	0.85

*PR: (Air Pressure/Liquid Pressure)
